# Helping Babies Breathe (2nd edition) implementation on a shoestring budget in Zanzibar, Tanzania

**DOI:** 10.1186/s40748-020-00117-z

**Published:** 2020-06-01

**Authors:** Gina M. Wilson, Ame M. Ame, Maimuna Mohamed Khatib, Bimkubwa Suleiman Khalfan, Julie Thompson, Jane Blood-Siegfried

**Affiliations:** Duke University, Durham, NC USA

**Keywords:** Birth asphyxia, Neonate, Newborn, Resuscitation, Tanzania

## Abstract

**Background:**

To assess the efficacy and viability of implementing Helping Babies Breathe, a neonatal resuscitation program for resource-limited environments on a small budget in two of the largest delivery centers in Zanzibar, Tanzania. The quality improvement initiative concentrated on training midwives, who directly care for neonates at birth on Helping Babies Breathe to address high rates of neonatal mortality secondary to birth asphyxia.

**Methods:**

The convenience sample was 59 midwives working in the two delivery centers of interest in Zanzibar, Tanzania. The train-the-trainer implementation strategy with repeated measures design was used to assess knowledge and skills at three time points. Observations were completed through supportive supervision of deliveries in both facilities. A budget was kept throughout the implementation.

**Results:**

Knowledge scores and resuscitation skills significantly improved and were sustained over a 6-month period of time, *Ps* < .001. 130 supportive supervision observations were completed. Eighteen times (14%) a baby did not cry at birth and needed intervention. All were appropriately intervened for and survived the Golden Minute. The budget for this implementation was 9015.50 USD. Considering in-kind donations and financial support by the Zanzibar Ministry of Health the bottom line cost was much lower.

**Conclusion:**

Results indicate that participants retained knowledge and skills over time and were able to translate these skills into clinical practice. This initiative provides an alternative approach to implementing Helping Babies Breathe, relying on a small budget, local leadership and government support.

**Trial registration:**

Not applicable.

## Background

Based upon current trends, it is projected that approximately 56 million children under 5 years of age will die between 2018 and 2030; half of these will be newborns [1]. Considering this, the reduction of neonatal mortality is of significant priority [1]. Globally, in 2017 approximately 36% of newborns died the same day they were born and close to three-quarters of all newborn deaths occurred in the first week of life [1]. Although neonatal mortality rates fell by 51% globally between 1990 and 2017, there are still many regions and countries that continue to be very high. Sub-Saharan Africa is one of those with the highest neonatal mortality rates in 2017 with 27 deaths per 1000 live births [1]. The United Republic of Tanzania, a sub-Saharan African country, reported neonatal mortality in 2017 of 19 deaths per 1000 live births [2]. The semi-independent Tanzanian archipelago, Zanzibar had a neonatal mortality rate in 2015 of 28 deaths per 1000 live births [3]. The main causes of neonatal deaths in Tanzania are birth asphyxia (29%), prematurity (25%), and sepsis (20%) [2].

Considering the most prevalent causes of death, attention needs to be placed on reducing deaths attributed to birth asphyxia. Helping Babies Breathe (HBB) is a perinatal resuscitation training program developed by the American Academy of Pediatrics (AAP) for resource-limited environments [4]. Several implementations of HBB have resulted in positive outcomes [5, 6]. One example is a two-year HBB study conducted in 8 Tanzanian hospitals that resulted in a 47% reduction in infant mortality within the first 24 h, and a 24% reduction in fresh stillbirths [6].

Acknowledging the need for intervention, global health initiatives can be expensive. A study was conducted with the focus of completing a cost-analysis of an HBB program implementation and initial follow up in a large region of Tanzania with attention towards evaluating the cost of national scale-up [7]. Total cost for initial training, equipment, and follow up visits in the Mbeya Region was $202,240.00 [7]. This study conducted 49 trainings with 1341 health providers, which is approximately 27 participants at each session [7]. The average cost per individual training was $4127.35 or approximately $150 per participant. Overall, it was concluded that HBB implementations are relatively low-cost with potential for high impact on neonatal mortality [7]. The question should be considered, can a smaller approach with an even smaller budget yield similar impact?

## Methods

A mixed methods repeated measures design was used in this quality improvement study. Data were collected through interviews and knowledge/skills scores before implementation, immediately following implementation, and 6 months after; data were also gathered through focus group dialogue and supportive supervision of real-time deliveries. The study received approval from Duke Medicine Institutional Review Board with a letter of agreement from the Zanzibar Ministry of Health, protocol number: Pro00063180.

### Setting

Zanzibar is an archipelago separated from mainland Tanzania by the Indian Ocean. The cluster of small islands is comprised of two larger islands: Unguja and Pemba. The training occurred in two of the largest delivery centers in Unguja, Zanzibar, Tanzania. These busy facilities are located in the city center and deliver upwards of 17,000 babies each year.

### Sample

The convenience sample consisted of 59 midwives and nurses involved in the delivery of infants at these hospitals. Participation was voluntary. A total of 55 participants considered themselves nurse midwives while four defined themselves as Registered Nurse Officers. Of the 59 participants, 37 were employed by one delivery center and 22 by the other. Four experienced Zanzibarian HBB master trainers in addition to the sample of 59 participants were selected in collaboration with the Ministry of Health to lead this implementation. The experienced master trainers had previously led successful HBB trainings in the Northern part of the island [8].

### Implementation

The innovation was comprised of two parts. The first was HBB training, a neonatal resuscitation program developed by the AAP for resource limited environments. HBB was created to be used by anyone responsible for neonates at delivery. The program’s guiding principle is that everyone can learn simple techniques to assist neonates who are unable to breathe on their own at birth [4]. Participants work together in small groups to practice key skills. HBB curriculum focuses on thermoregulation through skin-to-skin measures, stimulation to breathe, and assisted bag-mask ventilation if needed. Essential to the HBB curriculum is training participants to respond with the correct actions in the “Golden Minute,” the initial 60 s after birth [4]. During this period of time, caretakers have the highest chance of positively affecting survival.

The second part of the implementation was training midwives to conduct supportive supervision, and collect data that will help inform their practice, with the goal of completing quality improvement cycles. A birth record and quality improvement outline were developed in collaboration with local midwives. The birth record collected data regarding specific key processes consistent with the HBB Action Plan. The quality improvement outline aimed to help midwives use the data they were collecting to inform practice. They learned how to calculate percentages based on the key processes collected on the birth record. The next step outlined how to plot the percentages calculated each week on a run chart. The outline walked the reader through steps on how to perform this process weekly while looking for trends over time. The guide ultimately empowered nursing leaders through sharing these data with their team(s), brainstorming quality improvement efforts based upon identified areas of high need, and implementation of quality improvement measures to impact outcomes over time.

The implementation was uniquely designed to be completed on a small budget with increased financial and collaborative partnership with the Ministry of Health. The implementation took a grassroots approach in an effort to be more cost-effective and locally led with the overarching goal to be sustainable over time.

### Measures/procedures of innovation

The train-the-trainer model was used as the implementation strategy to complete this project. This entailed a certified AAP HBB instructor refreshing, training, and providing updates noted in the 2nd edition of HBB to the local HBB master trainers, then supporting the local HBB master trainers while they planned and trained other participants. Training included several different learning techniques, such as, hands on demonstration, return demonstration, simulation using NeoNatalie with different case scenarios, overview of HBB Action Plan, small group teamwork, as well as, recommended steps for assembling, disassembling and cleaning resuscitation equipment. Written materials included HBB 2nd edition Action Plan and Provider Guide.

The length of each training was 3 days. After initial training for master trainers, all four master trainers practiced and planned how they would deliver subsequent rounds of HBB trainings for 59 participants. Time was spent discussing HBB curriculum and master trainers practiced their delivery method of HBB content. Trainings were held in the two targeted delivery centers to increase accessibility for attendees. Each participant received a HBB learner handbook and was paired with another participant for hands on demonstration, return demonstration, and simulation scenarios. Both facilities were provided with upright bag-mask resuscitators, penguin suction devices, large HBB Action Plan posters, and NeoNatalies for ongoing practice opportunities.

Over a 6-month period immediately following the conclusion of trainings, master trainers completed 130 supportive supervision observations of real-time deliveries in both delivery facilities. The tool used by master trainers when completing supportive supervision was a one-page document created with the HBB Action Plan on one side, as well as, action steps correlated with the HBB Action Plan and curriculum to identify which steps were completed and which were not. The birth record also allowed the reviewer to note the baby’s outcome, such as, “alive - routine care”, “alive – special care”, “death during delivery”, “fresh or macerated stillbirth.” The form had a feedback section asking the reviewer what was good, what could have been better, and how to improve next time.

After each observation, master trainers reviewed forms with each midwife. Participants were also reminded about the importance of proper cleaning of resuscitation supplies between uses. Practice stations with a NeoNatalie and bag-mask resuscitator were set up in both facilities for midwives to practice as time allowed over the 6 months following initial training. The master trainers freely communicated with the AAP HBB certified trainer in the United States of America during these 6 months. Communication was completed via email and messaging apps.

The master trainers helped to develop and were trained on the quality improvement outline after initial implementation. The outline detailed the process of calculating percentages of HBB action steps performed during observations and how to create run charts to identify potential trends. This was to be completed weekly over the 6-month period of time to provide real-time feedback for clinical staff. During a one-day training midwives practiced using the quality improvement outline. Time was spent discussing how data could be shared with their teams to identify areas of need and create ongoing quality improvement efforts.

Six months after initial training, the AAP HBB instructor returned to Zanzibar. At that time, all 59 participants were invited back for a final evaluation of retention of knowledge and skills. The same measurements collected pre and post implementation were completed again. A focus group discussion was held by a non-affiliated third party to identify barriers, ideas for improvement in the future, and anecdotal information through storytelling of the past 6 months.

Throughout the implementation data were collected regarding the costs of completing this implementation.

### Data collection

Each participant was interviewed prior to implementation. This was completed to identify previous resuscitation training and availability of resuscitation supplies.

Five tools were used to assess participants HBB knowledge and skills and were repeated over three time points, pre-intervention, post-intervention, and again about 6 months after initial implementation.

- General knowledge of HBB was measured using multiple-choice questionnaires completed either written or verbally. The same 17-item questionnaire was used pre-intervention and post-intervention. This tool was an adapted knowledge check questionnaire incorporating HBB 2nd edition curriculum. A different 18-item questionnaire was used 6 months after initial implementation. This 18-item questionnaire was the HBB 2nd edition knowledge check questionnaire released by the Helping Babies Survive Program. These tools were very similar and a majority of questions used were the same on both tools. Both tools accurately asked questions based upon HBB 2nd edition content.
- Bag-mask skill performance was evaluated through demonstration of skills needed to perform bag-mask resuscitation. A neonatal simulator, NeoNatalie, was used to assess bag-mask ventilation technique and speed. Points were given for each skill correctly demonstrated. A score of 14 is the maximum possible score. The same evaluation tool from HBB 2nd edition was used at all time points.
- One objective structured clinical examination (OSCE) was used to assess the participants’ ability to demonstrate HBB skills in a simulated environment. Each participant was observed and points were given when skills were performed at the correct time and using the correct technique. A neonatal simulator, NeoNatalie, was used during this evaluation. The OSCE was validated as a single use OSCE for HBB [9].
- Supportive supervision was completed by master trainers observing HBB trained midwives conducting real-time deliveries between post implementation (time point 2) and 6 months after initial implementation (time point 3). The supportive supervision form was comprised of a checklist of action points that correlated to the 2nd Edition HBB Action Plan. The HBB Action Plan was part of the form and a section for feedback was included to define what was good, what could have been better, and how improvements could be made next time.
- A focus group was held 6 months after initial implementation. A member not associated with any trainings facilitated the group discussion. Open-ended questions were used to gather insight from trainees regarding satisfaction, identification of barriers related to translation of knowledge and skills into practice, ideas for improvement in the future, and storytelling of real-time deliveries in the past 6 months.

### Data analysis

Data were managed through Microsoft Excel. All data collected were de-identified by master trainers in Zanzibar, Tanzania prior to being shared with AAP HBB instructor.

Data from interviews were analyzed using descriptive statistics. Paired t-tests were conducted to compare pre and post implementation scores on knowledge, bag-mask skills, and objective structured clinical examination (OSCE) scores. Descriptive statistics (Mean, SD) were used to evaluate sustained knowledge and skills at the 6-month timepoint. Quantitative data were analyzed using IBM SPSS v. 25 with alpha set to .05. Data from supportive supervision observations were analyzed using descriptive statistics.

Data collected from the focus group were analyzed by reviewing the transcription of dialogue to identify themes. The focus group facilitator transcribed the dialogue. Transcriptions were reviewed to identify overall themes.

## Results

### Midwife interviews

Of the 59 participants, 37 (63%) worked in one main delivery center and 22 (37%) worked in the second delivery center. All participants worked clinically caring for mothers and neonates in the two largest delivery centers in Unguja, Zanzibar, Tanzania. Of the 59 participants, 55 (93%) considered themselves a nurse midwife and 4 (7%) considered themselves nurse officers. The majority (80%) of participants worked in maternity at their respective delivery center; while 10 (17%) worked in neonatal/kangaroo mother care and 2 (3%) worked in the theater. Everyone participated in the complete training duration.

Of the 59 participants, 21 (36%) stated they completed prior training on neonatal resuscitation while the majority (64%) reported they had not. When asked if the participants had access to neonatal bag-mask resuscitators at work 33 (56%) stated they did while 26 (44%) stated they did not.

Participants were asked an open-ended question about what barriers at work were interfering with helping babies who were having difficulty breathing at birth. 24 (41%) participants reported a shortage of staff and lack of equipment. 23 (39%) participants reported a lack of equipment. Together, 47 (81%) of participants reported either a shortage of staff, lack of equipment or both.

### Knowledge, bag-mask, and objective structured examination

Paired *t*-tests were conducted to compare pre and post implementation knowledge, bag-mask, and structure evaluation scores. When testing assumptions related to normality and outliers for paired t-tests, the difference between the paired values are examined, as opposed to the original variables [10]. The difference scores were normally distributed for all three outcomes according to Shapiro-Wilks tests. Specifically, difference scores for knowledge, bag-mask, and objective structured examination obtained Shapiro-Wilks *p*-values of .101, .273, and .313, respectively. Thus, we proceeded with paired t-tests.

Table 1 displays the results. As shown, all three outcomes had statistically significant improvement (all *Ps* < .001).

**Table 1:** Knowledge, Bag-Mask, and Objective Structured Clinical Examination Results

Outcome	***n***	Pre	Post	***p***-value
		Mean ± SD	Mean ± SD	
Knowledge ^a^	58	13.90 ± 2.02	15.64 ± 1.70	<.001
Bag-Mask ^b^	58	6.05 ± 2.87	12.84 ± 1.46	<.001
Objective Structured Examination ^c^	58	11.97 ± 3.69	18.02 ± 5.82	<.001

^a^Maximum score = 17; ^b^ Maximum Score = 14; ^c^ Maximum score = 23

Descriptive statistics (Mean **±** SD) were conducted at each timepoint (pre, post, and 6 months post implementation). Although two of the three 6-month outcomes were normally distributed (Shapiro Wilks ps ≥ .05), the median, min, and max are also presented. Available data showed that knowledge and skills were sustained after 6 months (see Table 2).

**Table 2:** Six Month Post Results

Outcome at 6 months Post Timepoint	***n***	Mean ± SD	Median (Min, Max)
Knowledge ^a^	21	16.29 ± 1.31	16 (14,18)
Bag-Mask ^b^	23	12.87 ± 1.18	13 (11,14)
Objective Structured Examination ^c^	23	19.13 ± 2.56	19 (14,23)

^a^Maximum score = 18; ^b^ Maximum Score = 14; ^c^ Maximum score = 23

### Supportive supervision observations

During the 6 months following training, 130 supportive supervision observations were conducted. Eighteen times (14%) a baby did not cry at birth and needed stimulation with one infant requiring suctioning. Thirteen times (10%) a bag- mask resuscitator was needed and used. In all 130 supportive supervision observations, all neonates began breathing and were able to independently breathe normally. All survived the Golden Minute.

In addition, other action steps that correlate with the HBB Action Plan were observed. One hundred twenty-nine times out of 130 observations the baby was dried thoroughly and the wet cloth was removed. Skin-to-skin was initiated 98 (75%) times and delayed cord clamping was completed 89 (68%) times. 84 (65%) neonates were left with their mother’s skin-to-skin after all interventions at birth.

The last component of the supportive supervision observations involved a discussion between the observer and midwife conducting the delivery to evaluate what went well, what could have been better, and how they could improve next time. See Table 3 for the most common responses reported.

**Table 3:** Reflective Portion of Supportive Supervision Results

“Went well”	Dried thoroughly	95%
	Removed wet kanga	88%
	Covered with a dry cloth	80%
“Could have been better”	Leaving baby skin-to-skin	36%
	Delayed cord clamping	28%
	Initiation of breast feeding	20%
“How we could improve next time?	Initiation of breast feeding	37%
	Leaving baby skin-to-skin after birth	21%
	Delayed cord clamping	16%

Total number of Supportive Supervision = 130

### Focus group

Six months after initial implementation focus group participants shared the following:

The most impactful parts of the training:

- Training was easily translated into practice
- The same equipment used in training was used in real deliveries
- Learned how to manage the Golden Minute using life-like simulations
- The HBB Action Plan made the steps easy to reference and follow
- Learning how to prepare the equipment and ventilation area was very helpful

Areas of improvement:

- Have a simulator that is a mother birthing a baby as part of the training
- Expand training for nursing students

Adjectives to describe how they felt after completing training:

- Proud
- Confident
- Happy
- Proud to be able to achieve the Golden Minute in training

Time was left open for midwives to share, as they felt comfortable, personal stories over the past 6 months when they used the skills learned in training. One story was shared about a midwife delivering twins. The midwife reported one baby cried at birth and was okay while the other had difficulty breathing and did not cry. The midwife reported she had prepared her equipment ahead of time and was ready to complete HBB steps. The midwife reported stimulating the baby to breathe and initiating bag-mask resuscitation in the Golden Minute. The midwife further explained that the baby began breathing after bag-mask resuscitation for 2–3 min. From those present at the focus group, the participants counted that they intervened for 15 neonates over the 6-month period of time.

### Budget

#### Data collection

The master trainers reported they were unable to complete the quality improvement outline. This included calculating percentages of HBB steps carried out during observations and plotting these percentages on run charts for 6 months following training. Barriers included staff shortage and patient care demands; however, they felt empowered and equipped to collect their own data, identify gaps in care, and work towards improving patient care. One master trainer stated “Nurses can collect their own data? Wow, this is great.” The budget for this implementation is reflected in Table 4.

**Table 4:** Budget

**HBB Implementation Budget - Zanzibar, Tanzania**	
**Trainer Transportation Expenses**	
Airfare	$1700.00
Lodging ($70/day)	$2100.00
Local Transportation ($100/week)	$400.00
Meals ($200/week)	$800.00
**Subtotal**	**$5000.00**
**Implementation Expenses**	
7 NeoNatalie Complete Training Sets with Upright Bag-mask Resuscitators (Dark)	$602.00
27 Upright Newborn Bag-Mask Resuscitators	$504.00
27 Penguin Newborn Suction Devices	$121.50
Freight Costs to Zanzibar, TZ (HBB Training Supplies)	$330.00
Training Snacks and Drinks ($30/training)	$120.00
Miscellaneous (ex: pens, printed materials, binders, name tags, etc.)	$100.00
**Subtotal**	**$1777.50**
**Partner Expenses & In-Kind Donations**	
*Trainee Transportation Fees (Covered by Zanzibar Ministry of Health)*	$1890.00
*Facility Rental (Covered by Zanzibar Ministry of Health)*	$200.00
*2 HBB Flip Charts and Posters*	$60.00
*80 HBB Provider Guides*	$88.00
**Subtotal**	**$2238.00**
**Total Project Costs**	**$9015.50**

## Discussion

### Key results

The following results support the objective to assess the efficacy and viability of implementing HBB on a limited budget using the steps of this project:

The results from the Knowledge, Bag-mask Skill Performance, and Objective Structured Clinical Examinations had significant improvements in all categories, immediately after implementation, and 6 months later. Improvements were maintained over time. Scores in all categories continued to increase during the 6-month period of time without additional trainings. There are many potential influencers of these results. Trainings were led by local leaders with prior experience teaching HBB in Zanzibar [8]. All master trainers were midwives delivering babies in the facilities of interest. Trainings were conducted in Kiswahili and were interactive as designed by the AAP HBB program. The train-the-trainer implementation strategy used has effectively been incorporated in other HBB studies [5, 6]. This strategy promotes ownership and empowerment of those being trained to lead and train others.

Another potential influencer of these results may be attributed to the 3-day duration of training. This increased timing was supported by comments from other HBB implementations, as well as a study that completed a one-day HBB training and resulted in skills not being translated into practice [5, 6, 11, 12]. Continued improvements in scores over time may also be attributed to midwives incorporating HBB knowledge and skill in every day clinical practice, supportive supervision observations by master trainers, and ongoing use of NeoNatalie practice stations setup in each facility.

The Supportive Supervision Observations demonstrate that HBB knowledge and skills were successfully translated into practice and midwives appropriately cared for 130 neonates. Furthermore, the observation data supports that midwives successfully intervened for neonates who had difficulty breathing at birth. The approach used during observations was non-punitive and incorporated shared decision making and skill development. Observations provided an opportunity for the midwife and the master trainer to reflect on what was good, what could have been better, and how improvements could be made next time. This reflective process is likely to have positively influenced care going forward by encouraging self-reflection as a valuable practice in midwifery training to foster ongoing self-awareness and professional growth [13].

Investment from local government officials was critical for the implementation of this project. All steps of the project were formalized in partnership with the Zanzibar Ministry of Health, as well as the implementation being written in their budget for the calendar year. Attention was placed on developing a reasonable budget that was practical and focused on items most likely to impact patient care. These efforts led to increased end-user buy-in and leadership, which influenced the overall success of this implementation and has increased the likelihood for this program to be sustained over time.

Local investment and leadership in quality improvement projects like this one is supported by other global health implementations that highlight the importance of involving local leaders [14, 15]. A systematic review of health interventions implemented in sub-Saharan Africa found that community ownership and mobilization were critical components for sustainability [15].

Global health programs are often measured on cost-effectiveness and direct health outcomes that can have downstream effects [16]. This implementation outlined a small budget that was cost-effective and positively impacted direct patient care. There has been a downstream affect from this implementation and an increased likelihood toward sustainability with funding partially provided by the local government. Joint programs are much more effective because they promote ownership of the content and increase sustainability efforts.

### Limitations

One of the limitations of this implementation is that not all participants returned for evaluation 6 months after initial training. All participants were invited to return, but fewer than half were able to participate. This was due to staffing constraints, participants being away on leave, and personal conflicts. The return and evaluation of all participants at 6 months post intervention would have provided a more complete review of knowledge and skill retention.

Supportive supervision observations conducted by master trainers may have resulted in implicit bias considering the approach taken in this study. During these observations midwives knew they were being observed by a master trainer that provided real-time mentorship and joint problem solving during deliveries. The midwife and master trainer completed the reflective process of the observation at the end of each delivery. Therefore, the results of the observations may have potential bias.

Another limitation is the portion of the quality improvement outline where clinical staff were to calculate percentages of HBB steps completed based upon supportive supervision assessments and plot the percentages on a run chart. These steps were not completed during the 6-month period of time. Barriers included staffing constraints and patient care demands. It would be beneficial to evaluate and determine the feasibility of this portion of the implementation and possible alternative methods to aid in its completion. Ultimately, continuous documentation of data from deliveries is needed for ongoing quality improvement.

Methodologically, increasing the sample size to allow for stratifying groups that had received some sort of neonatal resuscitation education prior to training compared to those who had not would offer more insight into the study. Thus, the results of the implementation may have been influenced by those who had received some form of resuscitation training in the past. The immediate test data were matched pre to immediately post, but the data 6 months later were not obtained on all participants, therefore the findings 6 months after initial implementation should be interpreted with some caution, as we are unable to assess each individual’s retention scores.

### Interpretation

This HBB implementation positively impacted care of neonates in the two delivery centers of focus in Zanzibar, Tanzania. The potential for replication in similar resource-limited environments is strong considering the key practical components and results that led to the success of this implementation. A team with a small budget, local leadership and buy-in will likely be able to yield a positive impact on care of neonates through a similar implementation of HBB in a like environment. Other HBB implementations with similar strategy have also yielded positive results [8, 12, 17]. The limitations are acknowledged and will provide a framework to build on going forward. This implementation is small and grassroots. Therefore, because of the nature of low cost programs like this scaling would be more difficult and perhaps not as effective. Strength comes from the presence of local leadership and buy-in, as well as, financial participation by the local government. Ultimately, the goal of moving towards sustainability and continuous quality improvement is formalizing a practical way for midwives to collect their own data of deliveries and this data to then be used to inform practice as a catalyst to further quality improvement efforts.

### Generalizability

Generalizability for this project is limited because it was not randomized and controlled research. An evidence-based program (HBB) was implemented with local midwives in Zanzibar. This quality improvement effort yielded positive results that have potential to be replicated in similar environments. This implementation provides a practical approach to improved care without large financial stewardship making it more accessible and sustainable in resource-limited environments; however, it is understood that each location is unique and these results may not be effective in other areas.

## Conclusion

This HBB implementation positively impacted care of neonates in the two largest delivery centers in Zanzibar, Tanzania. Furthermore, this initiative provides an alternative look at implementing HBB on a conservative budget relying significantly on local leadership and government. Overall, this quality improvement effort demonstrates HBB knowledge and skills were retained over time and translated into practice.

## Data Availability

Upon request.
